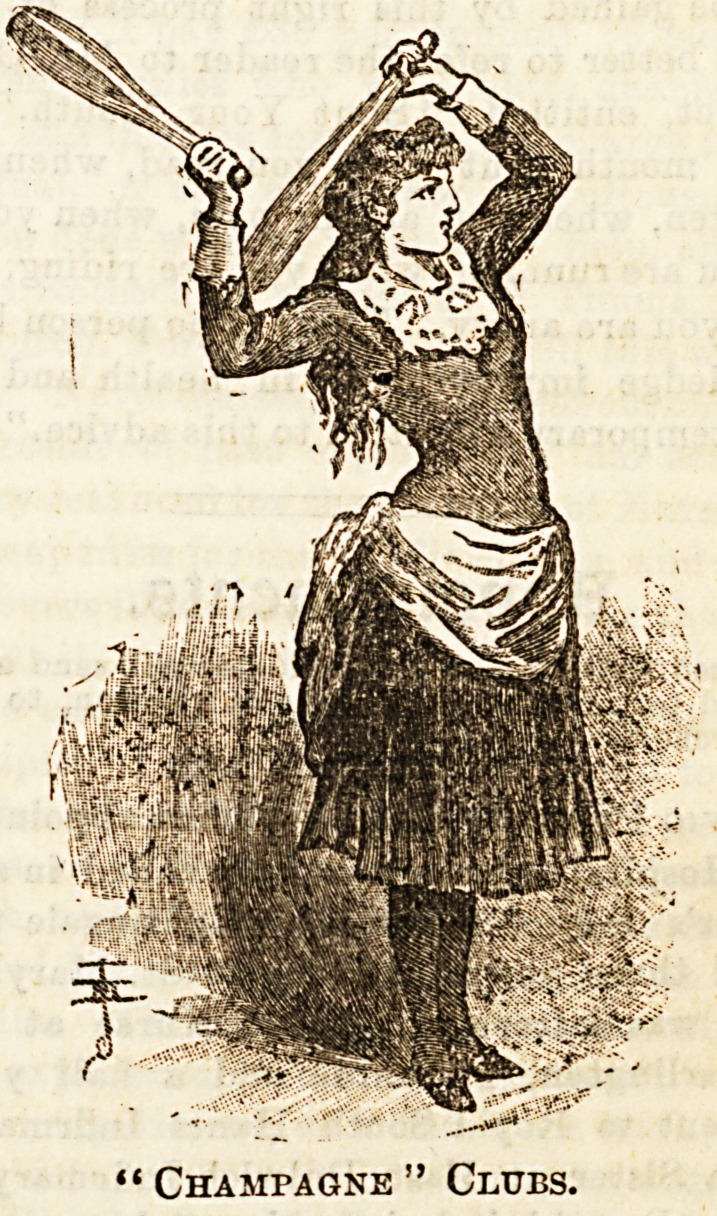# The Hospital Nursing Supplement

**Published:** 1893-01-14

**Authors:** 


### "The Hospital" Nursing Mirror.

Being the Extra Nursing Supplement of "The Hospital" Newspaper.

[Contributions for this Supplement should be addressed to the Editor, The Hospital, 140, Strand, London, W.C., and should have the word "Nursing" plainly written in left-hand top corner of the envelope.]

### News from the Nursing World.

### NURSES' CHRISTMAS HOLIDAYS.

"I suppose you all get holidays at Christmas, nurse?" is a remark not unfrequently made by a visitor in hospital wards. The person addressed must sometimes feel inclined to startle the stranger by replying, "Oh, yes, of course; and the patients take care of themselves." However, it is charitable to suppose that the speech is made from "want of thought," not "want of heart." Sickness is always unwelcome, but never more so than when the stricken patient watches from a couch of pain the death of the Old and the birth of the New Year. On many a future festival his own thoughts and those of his nearest friends will vividly picture the time when the interests of all were centered in that sick bed. With everything that skill and kindness can do to alleviate pain, sadness must always be present, but if the suffering only remained, and the careful attendance ceased, a pitiful climax would be reached. Surely no visitor need ever imagine that a nurse worthy of her profession would willingly desert her patient at any time, and no Christmas joys meriting the name could arise from neglected duties. Probably no nurses desire to receive any pity for spending the annual festival amongst those to whom they so freely give their services, and from whose bedsides they could be so ill spared.

### A CRITIC.

There is always amusement to be found in a crowd by a silent observer. At the opening of the Royal Eye Hospital at Southwark, whilst the audience which filled the Surrey Theatre awaited H.R.H. the Duke of Clarence, a complacent British matron certainly entertained her near neighbours. "Dear me!" she remarked, "what a number of them! All in uniform, too. Well, t'would be a deal better for the nurses to stop in their hospital, I should say! Not parading about in their uniforms." The worthy lady was ignorant that the group of comely women she criticised consisted of representatives from several large general hospitals, and they would certainly have outnumbered the patients had they constituted the staff of the little Eye Hospital. Moreover, the new building contained neither patients nor resident nurses. But the British matron maintained her severity. "Nurses, indeed!" she ejaculated at intervals, unconscious of the cordial reception accorded to each one of the quiet uniformed figures by most of the audience to whom the presence of the nurses was evidently gratifying.

### OUR ENDOWED BED.

Many grateful letters have been received from nurses who have reaped the advantages of the endowed bed. and who have returned to their work rested and refreshed by their much-needed holiday. This week we have two letters from subscribers, and we cordially agree to the suggested discussion in our columns. Nurse Annie's ideas about the journey to Ventnor deserve consideration; and F. L. E. is evidently an energetic and interested contributor. We are puzzled by her suggestion that "nurses taking part in the scheme should have the first right of occupancy," as she also names one thousand as the probable number of supporters. To think of so many persons all "having a right" to make use of the bed is alarming to the uninitiated, and such a plan must be carefully worked out by people having no personal interest in the matter, who would therefore judge fairly between conflicting claims.

### LOANED LOVELINESS.

Christmas festivities and thoughts, and even the special kindnesses which group themselves round the twenty-fifth of December, will soon be forgotten. At any rate, they will pass into the background of our memories, and lie there for twelve months to come. But in hospitals the performances do not end with the public display, any more than in private houses, where "putting away," or "getting straight," is an important completion of fête days. Some institutions are lucky in loans, such as flags, Chinese lanterns, pianos, &c., so that the expense of the decoration of the wards is considerably lessened for the Sister, whose outlay is confined to the breakages. But the "fairy lamps" and the cups and saucers are generally hired, and many other things of which the annual visitors know nothing. It would be well, perhaps, if the latter sometimes tried to learn how these things are done. Wherever free loans are made to the hospitals they are always gratefully acknowledged in the printed reports of the entertainments. To some of the wards at St. Thomas's Hospital most beautiful examples of Doulton's pottery were lent by the manufacturers. Costly columns surmounted by pots for palms, and smaller stands for flowers, all lovely loans, so in harmony with the position they held in lobbies and wards that it seemed a pity no one came forward to exercise such seasonable liberality as would prevent their ever going away again. We think that "getting straight" after their successful Christmas display would assuredly be attended by regret on the part of these favoured ward sisters.

### SUCCESSFUL SALE AT SHOEBURYNESS.

If our British soldiers carry all before them, certainly their wives and daughters are no less successful when they take in hand either work or play, or, better still, a happy mixture of both. There was a sale of work at Shoeburyness the other day, and a huge Christmas tree, in aid of the funds of the Garrison Women's Hospital, which, not being supported by Government, wants a great deal of voluntary pecuniary assistance. Many of the useful and ornamental articles exhibited were made by the wives of officers and soldiers, and Mrs. Richardson organised a working party, which accomplished a quantity of capital contributions. The Commandant, Colonel Richardson, lent the theatre for the occasion, and it was crowded by military and civilian spectators, who proved themselves also eager purchasers. A great deal of steady, hard work was certainly rewarded by this excellent result, and "play" was represented on this occasion by two hours of pleasant and spirited dancing after the sale had been concluded, and every article disposed of.

### WITH EMPTY HANDS!

The matron of the Ipswich Nurses' Home has certainly solved the problem of helping the helpless effectually. But she wants donations to enable her to enlarge the field of her nurses' useful labours. In visiting the sick they find coals, blankets and nourishment are as urgently needed as the steam kettles and other minor necessities. And, so far as they can, they supply what is wanted, but, alas, we read with regret in this practical matron's letter, "I should be thankful for more subscriptions, as I am obliged to refuse the least severe cases simply for want of funds." And so "the least severe" have to be let alone, with the risk that minor ailments, which might be cured by immediate attention, develop into illnesses of serious dimensions. But, as Miss Pye remarks, "an empty-handed nurse cannot do much in a cottage, especially at this season, when so much is needed."

### HALF-HEARTED BENEVOLENCE.

The committee of the Bradford Nurses' Institution have recently decided to raise the salaries of their workers. Instead of giving £25 for the fifth year of service they promise that amount for the fourth, and agree to an annual increase until the maximum rate of £31 is attained. The district nursing is arranged on a curious system at Bradford, for the townspeople's subscriptions are only sufficient to cover half the expenses of the three trained nurses whose services are most gratefully appreciated by the sick. Most promoters of schemes for nursing the poor in their own homes begin by "counting the cost," and then they take steps to raise the money required. Perhaps the Bradford people wish to figure as the historic exception which goes to prove the rule. Whatever their motives, the result is unlucky, for the earnings of the private nurses go to make up the existing deficit. For the fair gains of one set of nurses to be absorbed in defraying the cost of another contingent is surely an arrangement which ought to be scorned by the liberal citizens of Bradford. It is by no means the custom of the kindly North-country folks to dispense in charity that which has cost themselves nothing.

### A WELSH MINISTER'S "CURSE."

The Bangor and Beaumaris Guardians have been wisely considering the subject of a trained nurse for their infirmary. Mr. David Williams observed, in support of the motion, "that a trained nurse was the proper person to look after a hospital"—an assertion which could hardly be gainsaid. However, the Rev. Mr. Jones thinks otherwise, evidently, and we regret to say, in his experience, "a trained nurse was the greatest curse that ever entered a house." Poor Mr. Jones!—or should we say "lucky Mr. Jones"? In the first case, he needs sympathy for his unhappy "experience," but in the last we incline to congratulate him on the grounds that his knowledge of "curses" must be limited if a nurse is the greatest one he knows. If he had mildly said "some" nurses, we could have let the assertion pass, but we really must venture on a challenge when the whole of the noble race is thus publicly denounced. To meet the wishes of this original minister, a resolution was passed by the Board that the advertisement should say "assistant matron" instead of "trained nurse," as originally proposed. Perhaps the alteration is a wise one, as the first may possibly be attainable, but certainly the latter can seldom be procured "at a salary of £18 per annum"!

### MATER MISERICORDIÆ HOSPITAL, DUBLIN.

We are pleased to hear that the new system of nursing recently adopted at this well-known hospital is working satisfactorily. The sisters have not only installed a trained nurse as teacher of the probationers, but they, and all members of the staff, are working cordially together in the endeavour to establish and to maintain a really good school. They have instituted examinations, in which the medical staff have shown an unusual amount of interest, and we have every confidence that a work so well begun will end prosperously.

### PLEASED PATIENTS.

The patients in the South Infirmary at Cork seem to have had a good Christmas, and to have been entertained with an excellent concert and a magic lantern display at the recent festive season. This, happily, is the pleasant portion of many sick inmates at many institutions now that intelligent attention is devoted to the subject of pleasure, as well as to that of pain. But the novelty attaching to the entertainment at Cork lay in the scene of the show, for this was the Operating theatre. Those present on the occasion will in future retain a curious medley of emotions, for instead of a sigh or a shudder at the mention of the room, a retrospective smile will appear on many a face wasted by sickness. Decorations and mirth banished for the moment thoughts of sadness, past or future, and the company assembled gave and received true Christmas pleasure. It is a scene worth dwelling on, from its unusual character. We are more accustomed to hearing fanciful visitors shrink away from the door on which they see the inscription of "Operating theatre," with a nervous glance at the threshold lest some horror declare itself there! But the patients at Cork, looking neither backward nor forward, enjoyed the good things of the present, and rewarded their entertainers by their hearty Christmas rejoicings.

### AN AUSTRALIAN NURSES' HOME.

The new Nurses' Home, in connection with the beautiful Prince Alfred Hospital at Sydney, was opened on December 13th by the Countess of Jersey, and we hope soon to give our readers a detailed description of the buildings as well as of the ceremony. So fine a hospital certainly merited an equally perfect Nursing Home.

### SHORT ITEMS.

Nurse Elms has sent us half-a-crown for the endowed bed, and Nurse Shore one-and-sixpence.—Miss Carty's Nursing Institution is now established at No. 2, Bulstrode Street, Welbeck Street.—The Herts and Canterbury Nursing Institute are giving considerably larger salaries than formerly.—We beg to acknowledge with thanks twenty shillings for the endowed bed from W. Parker.

### The Development of Children by Gymnastics.

### IV.—INDIAN CLUBS.

THE last exercises of our first series with movable apparatus are those with Indian clubs. These are perhaps the best suited to children, and girls in particular, being, of course, performed by them with very light clubs, for these of all others require the most "finish," and tend to give the ease and grace which are essential to every lady.

The general effect to the spectator, and, indeed, the pleasure to the pupil, is greatly enhanced by musical accompaniment. Most of the exercises are performed to a moderately slow waltz tune, which helps the easy swing of the clubs, contrasting so favourably with the uncouth jerks introduced by many, and especially children, if unaccompanied by music. From earliest times gymnastics have been closely allied with music. Competitions in singing were introduced as far back as the Pythian games, and Plato is reported to have said that "the best gymnast is sister to pure and simple music. By the one health is given to the body, and by the other self-control to the mind, so that both together form a complete education. Gymnastics alone, or music alone, produce effeminacy." But we must here venture to introduce, according to our more modern notions, a third thread to form the perfect or "complete" cord of education: the mental training of the brain apart from music; but which, together with the arts, prevents the gymnast from sinking to the level of the brute athlete, who was looked on by Hippocrates as "soulless."

In many of our gymnasiums, whistling, on the part of the men, is combined with the use of the dumb-bells as a means of further exercising the lungs; this, in effect, answers no doubt much the same purpose as instrumental music so far as the rythm is concerned, but it is a greater tax on the gymnast. Many teachers condemn the practice of musical drill on the score of its dividing the attention of the pupils, and therefore detracting from the advantage of the exercises; but we do not see how this objection can be weighed against the consideration of the immense assistance afforded by music to the correct and finished movements of the various exercises, and especially when they are performed in class, since it helps the children to work together. The great evil to be avoided is, that the pupils should acquire a habit of slurring over the exercise, so that any one movement is lost in the endeavour to "catch up" with the music. Whether this does or does not follow, of course, depends wholly on the vigilance of the teacher; and, it may here be noticed, that it is partly owing to the far greater importance attached by the generality of instructors of the Swedish drill to the exact performance of the slightest detail, that the Lyng system has produced a larger majority of satisfactory results than any other school of physical education.

In selecting a suitable club for children it is well to choose one rather slighter in shape than the Champagne—those shown in our illustration—as the weight is then farther from the handle, and this gives more leverage, making the swing easier, and at the same time leaving the end better adapted to the grasp of small fingers.

Of course, the single heavy club is out of the question as far as young pupils are concerned, and should be altogether avoided by girls, as a strain far too great for their muscles, which, it must be remembered, are even more injured by over-work than by an undue proportion of repose, especially when the body is growing.

The ordinary Indian clubs serve to boys as a gradual preparation for the more arduous exercises, with heavy weights, which come to them later in the gymnasium course, and which, without the milder, introductory calisthenic course, would probably do more harm than good.

The chief muscles called into play are those of the arms shoulders, and legs; and in some of the exercises we have almost all the muscular system more or less in action. It is almost impossible to convey a clear idea of the manner of performing the complicated movements of the clubs except by copious illustrations, demonstrating each stage of a single exercise; five minutes practical instruction is worth as many pages of explanatory print. Writers have, however, undertaken to show that it is possible to explain them on paper, of whom one of the clearest, and certainly the most original in plan, is "Lemaire." He explains at length many of the most complicated exercises in his treatise on "Indian Clubs and How to Use Them," the figures seem at first sight hopelessly involved, but careful attention to his explanation soon makes each movement appear easy. We may here give one hint which will be useful to remember—the clubs must always be regarded as moving in a plane. If the circles made by them are in front of the body, the planes are perpendicular and parallel to one another, and great care must be taken to keep each club moving in its own plane of action, as care-

"CHAMPAGNE" CLUBS.

lessness on this score often brings its special punishment, and that at times is severe. It is, perhaps, hardly advisable to warn pupils to be "graceful" in their movements, as true grace comes natually if the exercises are done with due attention, but unless the movements *are* easy, the clubs were better left untouched.

Let us here say a few words about the management of the breath during these and indeed all exercises. This is often left to be regulated according to the will of the pupil, and in consequence we see many panting and otherwise showing signs of loss of breath after some of the more vigorous movements. This, of course, comes from ignorance as to the proper way to breathe, a fact hardly to be credited in those whose very life depends on breathing. When inhaling, the lungs should be fully expanded and filled with air drawn in through the nostrils, not through the mouth, as is often the case. When exhaling the lungs should be emptied. We say filled and emptied, because the air with which we fill our lungs is largely life-giving oxygen, and we need all we can get of it; while that which we empty from our lungs is heavily laden with poisonous gasses of which we are well rid; therefore empty the lungs and refill them with pure air, only to be obtained through the nostrils, where nature has provided us with a perfect air-filter. Those who know anything about the atmosphere by which we are surrounded, know well that it contains many impurities injurious to the lungs; now these are removed by the minute hairs which line the nostrils, and the air is thus prepared to be received into the lungs, whereas, when drawn through the mouth, it is charged with all sorts of organic matter, which often breeds disease, and materially shortens life. It will be found a great advantage to have acquired this habit when young, and learnt in connection with drill; it teaches us to inhale and exhale regularly, as also to husband the store of breath when taken; this is most essential in running, rowing, walking, talking, dancing, singing, and reading. When correcting children for "gasping" as they read, we have often been met with the prompt answer, "Oh, I always breathe through my mouth, I can't do it any other way." This is, of course, absurd; they can, though a bad habit once acquired is most difficult to overcome. It is, however, quite worth the trouble necessary, and in many cases it will be found that once the breath has been mastered, reading—and even singing—will become fluent and intelligible, since the mouth is left free for utterance. One might write at much greater length on the advantages to be gained by this right process of respiration, but it will be better to refer the reader to Mr. Catlin's book on the subject, entitled, "Shut Your Mouth." He says, "Keep your mouth shut when you read, when you write, when you listen, when you are in pain, when you are walking, when you are running, when you are riding, and by all means when you are angry. There is no person but will find and acknowledge improvement in health and enjoyment from even a temporary attention to this advice."

### Appointments.

[It is requested that successful candidates will send a copy of their applications and testimonials, with date of election, to THE EDITOR, The Lodge, Porchester Square, W.]

MISS HEDWIG PROSCHIVITZKY has been appointed Sister at the Greek Hospital, Alexandria, and sailed in s.s. *Arcadia* on New Year's Day. She was a Nightingale probationer, and received three years' training at St. Marylebone Infirmary. She was afterwards Head Nurse at the General Hospital, Darlington, for three and a half years. From there she went to Royal South Hants Infirmary, and was afterwards a Sister at East Dulwich Infirmary for sixteen months. Miss Proschivitzky's testimonials are excellent, and we wish her every success in her new work.

### Minor Appointments.

[We propose to insert from time to time under this heading the appointments of Assistant Matrons, Charge and Staff Nurses, if our readers will kindly keep us informed of their promotion.]

PEMBROKESHIRE AND HAVERFORDWEST INFIRMARY.—Nurse Elizabeth Lloyd has been appointed Sister at this infirmary, and on Saturday December 24th, was awarded a silver medal (gift of the Matron) for excellent work done during the last two and a half years. Miss Lloyd was trained at Hereford General Infirmary, has been staff nurse at Pembrokeshire and Haverfordwest Infirmary for nearly three years, and has won the esteem of all who know her.

### Wants and Workers.

"A. M." WILL be glad to hear of a Home (free) for a boy blind from birth and suffering from water on the brain. He is five years old and quite helpless.

AN earnest appeal for warm clothes of all kinds is made by Nurse M. Moyoh, District Nursing Association, 82, Victoria Street, Ashton. The distress, following on the strikes, is very great. Nurse Moyoh thanks our readers for help given to her patients on previous occasions.

### Christmas Festivities.

CHARING CROSS HOSPITAL.—The cheerful wards were prettily decorated for Christmas, and the students of the hospital gave their fourteenth annual entertainment to the nurses and patients. There was a giant Christmas tree covered with gifts, and everybody enjoyed the various amusements provided. "Poor Pillicoddy" and "The Captain of the Watch" were admirably acted, the costumes being lent for the occasion by Messrs. Nathan, of Bear Street, Leicester Square, and the decorations were kindly supplied by Messrs. Defries and Back, of Covent Garden.

CHELSEA HOSPITAL FOR WOMEN.—A hundred articles of clothing were sent by the London Guild as gifts to the patients at Christmas, and the Ladies' Committee and other friends contributed in presents or in money towards the due observance of the festival. The patients were regaled with turkey and plum pudding on Christmas Day, and a very pleasant evening was afterwards spent.—The nurses' concert took place at the hospital on the 7th inst., when a very enjoyable evening was spent. The board-room was filled with the nurses and their friends, the convalescent patients, a number of lady and gentleman visitors, and members of the medical staff. Mrs. Frederick Davies, of Lexham Gardens, W., kindly took charge of the arrangements, and gave several recitations. The following artistes gave their services: Madame and Signor Mèo, Signorina Oliva Mèo, Mrs. Chase Marshall, and Miss Senior; also Mrs. Daunt, Mrs. Sutherland Morris, and Mr. Schank very kindly contributed solos. The farce, "A Pair of Lunatics," was given at the end of the programme, the parts being ably sustained by Miss Duff Bruce and Mr. Warren Melhuish. At the instance of Drs. Leith-Napier and Fenton, a vote of thanks was passed to Mrs. Davies and her company for a really excellent performance.

CITY HOSPITAL FOR INFECTIOUS DISEASES, NEWCASTLE-UPON-TYNE.—Special Christmas fare was provided in each pavilion for the patients who were well enough to partake of it on December 24th. In the scarlet fever ward most of the inmates were sufficiently convalescent to spend a thoroughly happy day. A magic lantern exhibition was followed by some very good amateur acting, in which both nurses and patients distinguished themselves.

ISLE OF MAN HOSPITAL.—The decorations at this hospital were exceptionally beautiful, and reflected great credit on the willing fingers of many busy workers. The corridor was universally admired and was most artistically arranged. The patients received capital gifts of a useful character through an imaginary parcels post. An excellent dinner was provided on Christmas Day, and the annual entertainment took place on the Feast of St. Stephen.

NATIONAL HOSPITAL FOR DISEASES OF THE HEART AND PARALYSIS, Soho Square.—The entertainments at this hospital comprised a Christmas tree on Christmas Eve, followed by songs, recitations, and some excellent carol singing by the choir of St. Anne's, Soho Square. Next day the patients had their usual Christmas dinner of turkey, plum pudding, and mince pies, followed by dessert. On New Year's Eve the Kilburn Choral Society gave a very enjoyable concert to the patients and their friends.

ROYAL INFIRMARY, PRESTON.—Christmas Day was made a very happy one at the infirmary, "thanks to the kind officials and donors of good gifts." Roast beef and plum pudding were followed by tobacco for the men and snuff for the female inmates. The chapel was admirably decorated, and so were the wards, fairy lamps and lanterns being here, as in many general hospitals, a specially attractive feature this year. The annual entertainment took place on January 2nd, and the children's ward received immense admiration.

Royal Hants County Hospital.—Christmas Day was ushered in by the singing of carols in the corridors and wards of the hospital, and the nurses and maids afterwards received warm thanks from the patients for this pleasant surprise. A sumptuous dinner was supplied by the liberal committee and was heartily appreciated, and in the evening the wards were lighted and thrown open to visitors, who greatly admired the tasteful decorations.

St. Mary's Hospital, Paddington.—Neither patients nor nurses have been forgotten at St. Mary's during the festive season, and the resident medical staff especially have been most commendably active in their efforts to cause pain and suffering to be forgotten. In the Children's Medical Ward the boughs of a huge Christmas tree were laden with presents for the little ones, the nurses, students, and hospital officials. The presents were distributed by the most gigantic Father Christmas, who appeared out of a box marked "A Case for the Children's Medical Ward." Many of the presents to the staff conveyed good-humoured allusions to the little weaknesses of the recipients, which caused much amusement. The balcony adjoining the ward was tastefully decorated, and converted into a tea-room for the guests, and was presided over by the nurses. Later in the week some excellent theatricals were given, the *dramatis personæ* being entirely composed of the resident medical staff. The female characters in the pieces caused much amusement. All the parts were well rendered, Tupper, represented by Mr. Symes, in "Chiselling," being really admirable. An excellent concert was given between the pieces. The patients, many of whom wore most artistic head-dresses over their bandages, appeared to appreciate greatly the efforts to amuse them.

Wirral Children's Hospital.—The little people who could not go home for Christmas Day had a capital dinner served to them in the prettily-decorated wards. The nurses had their annual supper on the following night, and on Wednesday afternoon the entertainment and distribution of presents from the beautiful tree took place.

### The International Nursing Congress, Chicago.

As stated in our issue of the 3rd ult., an International Congress on Nursing will be held at Chicago, beginning June 13th, 1893. It originated in America, and it is mete that the Congress should be organised by, and that the supreme control should be vested in, United States citizens. We are happy to be able to announce that the Chairman of the Congress will be Dr. John S. Billings, of Washington, the most eminent authority in the United States. It is further satisfactory to know that Dr. Hurd, of the Johns Hopkins Hospital, has been appointed Secretary, and Miss Hampton Chairman of the section relating to nurses. No better selections could have been made, and everyone in this country may now rest assured that the whole of the proceedings will be conducted upon the best lines, and that the Congress is worthy of, and should receive, the cordial co-operation of all British and Colonial institutions. A general invitation to send representatives will be issued to each large English hospital, and each will be asked to present papers, and to co-operate in other ways. The matrons and authorities of the nurse training schools will also receive an invitation from Miss Hampton shortly. Meanwhile, we shall be glad to answer any inquiries on the subject, and to hear from anyone who is anxious to contribute a paper, or to take an active part in the proceedings of the International Nursing Congress.

### Everybody's Opinion.

[*Correspondence on all subjects is invited, but we cannot in any way be responsible for the opinions expressed by our correspondents. No communications can be entertained if the name and address of the correspondent is not given, or unless one side of the paper only be written on.*]

### "NURSES AND THEIR FEES."

Sir,—Following on the letters from "A Patient" and "Ex-Nurse," perhaps you can find room for a few remarks from me on the difficulty of *supplying* skilled nurses to the middle classes and those in reduced circumstances. It seems to me that "Ex-Nurse," while advocating the nurses' co-operation system for this purpose, shows conclusively its impracticability, which she illustrates by an instance of a co-operative nurse's attempt in this direction. Nurses' training costs so much, and the work is very often arduous and trying, so that they should be sufficiently well paid to enable them to put by for sickness and old age. Two years ago I started an institution, which I commenced with nurses working on the co-operative system, paying 8 per cent. on their earnings, and 15s. per week including laundry when living in the home, but such nurses could not afford to nurse cases on reduced terms, and I therefore engaged some salaried nurses (to whom I pay £30 per annum) to meet the many demands from families who were quite unable to pay the regular fees, and in some instances the charge has had to be merely nominal, or else altogether gratuitous. This could not have been done without some kind help, and I have to thank Messrs. Rothschild, the Rev. J. H. Timms, and the Vicar of Christ Church, Forest Hill. But still this work does not pay its way. Nevertheless, I am endeavouring to carry it on, for as a result of past experience I am convinced that there is a very real need for the provision of Nursing Homes at relatively cheap rates for the middle classes, and also of nurses at home in severe illness at much lower rates than are usually charged. But it appears to me that this provision can only be wisely and effectually made by some extension of the provident principle. I therefore propose to add to the present accommodation of my house by furnishing a large room as a ward for persons of limited means, at a minimum charge of 15s. per week, and also to supply trained nurses outside at the low rate of 10s. per week in return for an annual subscription of 10s. These charges must necessarily be open to revision as experience points out the need, owing to the novelty of the experiment, as I think the work, when fairly started, should be made entirely self-supporting. The Hon. Mrs. Stuart Wortley has kindly promised her help, and I am in the midst of forming a committee who will relieve me of all further responsibility.—I remain, yours faithfully,

M. Robinson,

Lady Superintendent South London Nursing Institution, Forest Hill, S.E. (late Lady Superintendent Kent Nursing Institution, West Malling; and Matron of the Bucks General Infirmary, Aylesbury, and of the Suffolk General Hospital, Bury St. Edmunds).

### OUR ENDOWED BED.

Probationer Jane writes: "Will some experienced nurse please say whether it would not be nice to have our endowed bed named more definitely? Some title like 'The Hospital Endowed Bed' would please a great many regular readers of the paper."

"F. L. E." writes: "Now that funds are again being collected for the support of our 'endowed bed,' it seems to be a good time to submit to your readers the following plan, viz., to get an endowment in perpetuity by nurses co-operating in the matter. If a thousand of them could undertake to collect 30s. each by September next we could then invite a suitable committee to carry out the scheme. Probably subscribers would prefer giving contributions to a definite end, instead of being appealed to annually. We could in the meantime discuss the matter in this column of THE HOSPITAL, which is so kindly placed at the disposal of nurses. If a room were rented in a private house, the seaside town patronised need not always be the same one, if the committee deemed a change of locality desirable. Nurses taking part in the scheme should have first right of occupancy." *** We shall be glad to receive the names of those who are willing to collect a minimum sum of £1 and upwards for this bed. —ED. *T. H.*

NURSE ANNIE writes: "Our endowed bed is now again being talked about. We are all well pleased that THE HOSPITAL has been the means of starting such a useful plan, and we want to know if a change of locality would be welcome or unwelcome to the majority of subscribers? Ventnor is certainly not easily accessible, except to dwellers in the south, for whom it naturally has less attraction than for those who live in bleaker parts of England. If nurses would write their views on the matter, we could discuss it in THE HOSPITAL, as the Editor seems always willing to forward our wishes."

### NOT A POLICY-HOLDER.

"A READER" writes: "The R.N.P.F. has been freely criticised during its short and prosperous career. I have just met with such a sad instance of a sufferer who might have been relieved by sick pay or by the benevolent fund if she were but a policy-holder. She injured her back very seriously in helping to move a heavy patient under exceptionally difficult circumstances, and has been very ill for a long time now. The only way in which she has been able to make a little money has been by selling useful knitted articles made between her attacks of pain. That she is very ill there can be no doubt, and the doctor is attending her twice a day. She has various friends, but they are not well off, though they nurse her kindly and well. The knowledge that now it is out of her power to help herself, much less to earn her own living, is indeed bitter. She seems a woman who cannot ask help for herself, and yet her previous good work and good character would certainly entitle her to aid if she had been in days of health a subscriber to the R.N.P. Fund." *** We shall be glad to receive any contributions for this case, which our investigations prove to be one for sympathy and prompt pecuniary help.—ED. *T. H.*

### Presentations.

ON Christmas Day the pupil nurses presented Mrs. Phillips, Lady Superintendent of Queen Charlotte's Hospital, with a handsome silver-mounted dressing-bag, and a card with the name of the donors printed on it.

MISS WILKIE, Lady Superintendent of St. Luke's Hospital, Halifax, received a handsome afternoon tea service from her nurses upon Christmas Day.

ON Friday, December 23rd, the members of the Nurses' Residential Club, in Charlotte Street, presented the Hon. Secretary (Miss Cadenhead) with a pretty fern case; and the Manageress (Miss Rose) with some charming autotypes.

MISS DE PLEDGE, Matron of Chelsea Infirmary, received a charming combination card case and purse of silver from her nurses at Christmas.

THE nurses of Hope Hospital, Warrington, gave the Medical Superintendent (Dr. J. G. Gornall) a handsome serviette ring at Christmas, and they also presented Miss Whitaker (the matron) with an elegant brooch of pearls set in gold.

THE general staff of the South-Eastern Hospital presented the Rev. J. Mylins, the Chaplain, with a carved oak chair and reading desk on Christmas Day. Miss Ambler Jones, the Matron, received from the nursing staff and servants a handsome travelling bag, a Queen Anne's tea service in case, and a pretty bamboo table.

MISS ALEXANDER, Matron of Paisley Infirmary, was presented with a handsome silver inkstand by the nurses on Christmas morning.

ON New Year's Eve, the nurses of the Union Infirmary, Fir Vale, Sheffield, presented Miss Thomlinson, the Lady Superintendent, with an ornamental travelling clock.

THE nurses of the Blackheath and Richmond Nursing Institutions have presented Mrs. Smith and Miss Duncan, the Superintendents, with a handsome salad bowl and set of fruit spoons.

THE Nurses of the James Murray Royal Asylum, Perth, have presented the Matron, Miss Beatrice Mountford, with a handsome polished oak writing case, in token "of their love and esteem."

MISS ROGERS, who has resigned her position as Lady Superintendent of the Cornwall Trained Nurses' Home, was presented on her retirement with a silver-mounted purse containing twelve guineas. The Mayor of Truro handed the gift to Miss Rogers as a "practical recognition of the noble work she has done among the sick poor of the city." The money had been collected by Mr. John Edwards from a number of Miss Rogers' grateful friends.

THE nurses of the Glasgow Sick Poor and Nursing Association have presented a handsome silver tea service to their Superintendent, Miss R. Wood.

### Where to Go.

FOUR free lectures on "Inebriety and Jurisprudence" will be given by Dr. Norman Kerr at 11, Chandos Street, Cavendish Square, on January 10th, at twenty minutes past five, and on January 17th, 24th, and 31st, at four o'clock.

FREE lectures on "Physic," at Gresham College, Basinghall Street, by Dr. Symes Thompson, will be given at six p.m. on January 17th, 18th, 19th, and 20th.

### Notes and Queries.

### SPECIAL NOTICE.

The contents of the Editor's Letter-box have now reached such unwieldy proportions that it has become necessary to establish a hard and fast rule regarding Answers to Correspondents. In future, all questions requiring replies will continue to be answered in this column without any fee. If an answer is required by letter, a fee of half-a-crown must be enclosed with the note containing the enquiry. We are always pleased to help our numerous correspondents to the fullest extent, and we can trust them to sympathise in the overwhelming amount of writing which makes the new rules a necessity.

### Queries.

(4) *Dispensing.*—I would be very grateful for names of books suitable to study of dispensing.—*An Exile.*

(5) *Mental Nursing.*—I want to know where I can enter as paying probationer, and gain certificate on this subject. I applied to Berrywood, but have had no reply.—*Emerald.*

(6) *Beds.*—Can anyone inform me what kind of beds are in use at Guy's Hospital? Would they be good for small wards?—*Sister Amelia.*

(7) *Training in Liverpool.*—Where can I get trained as a general probationer in or near Liverpool? R.C. not objected to.—*A. M. G.*

(8) *Paying Probationer.*—Can anyone tell me whether they take paying probationers at the Royal Free Hospital, and if there is a Nurses' Home.—*Wilhelmina.*

(9) *Open Days.*—I want to know whether there is a general practice of showing the nurses' rooms on fête days at hospitals? At the Temperance in Hampstead Road. at the New Hospital for Women, and at the London, visitors were allowed to inspect every department. Is this usual?—*Rita.*

(10) *A New Departure.*—Can you tell me whether any hospital in London has only one bath for nurses and servants?—*Fanny.*

(11) *How to Become a Nurse. By Honnor Morten.*—Is this book reliable and up to date?—*Janet.*

### Answers.

(4) *Dispensing* (An Exile).—You will find all information you want in THE HOSPITAL newspaper of September 24th, 1892. under "How to Become a Dispenser." The new edition of Burdett's "Hospital Annual" will also give a chapter on this useful subject.

(5) *Mental Nursing* (Emerald).—Write again to Berrywood. Perhaps your letter has gone astray, for the officials are far too courteous to leave it altogether unanswered.

(6) *Beds* (Sister Amelia).—Write to steward or secretary at Guy's Hospital for information, which will be willingly given. We advise your reading two articles in "Workshop" of THE HOSPITAL newspaper issued on December 3rd and 17th, 1892, on the subject of bedsteads.

(7) *Training Liverpool* (A. M. G.).—Write to matrons at following addresses, and ask your question regarding the religious question when you apply, which will save you eventual disappointment: Northern Hospital, Royal Infirmary, Mill Road Infirmary, Royal Southern Hospital, Stanley Hospital, all at Liverpool.

(8) *Paying Probationers* (Wilhelmina).—Apply to the Matron. Paying probationers are taken, we believe. We know that a scheme for improving the accommodation for the nurses is under discussion.

(9) *Open Days* (Rita).—It ought to be a rule without exceptions that every institution supported by charity can be freely inspected in every department at all reasonable hours. Unfortunately, this is not the case, and at some places admission to even a part of the buildings is granted with grudging discourtesy.

(10) *A New Departure* (Fanny).—We hope that such a proceeding would not be tolerated in any hospital, although there is certainly only one very small bath at the New Royal Eye Hospital, Southwark, and the dining-room of the nursing staff is the recreation-room of the servants.

(11) *How to Become a Nurse. By Honnor Morten. Janet.*—The *British Medical Journal*, in its review of this book, said: This book of Miss Morten's must prove a perfect Godsend to those who are frequently appealed to as to the steps to be taken to become a nurse. We would advise everyone aspiring to be a nurse to consult the book before finally deciding what hospital or maternity institution she elects to train in.

## Figures and Tables

**Figure f1:**